# Anaemia among Female Undergraduates Residing in the Hostels of University of Sri Jayewardenepura, Sri Lanka

**DOI:** 10.1155/2014/526308

**Published:** 2014-09-22

**Authors:** Gayashan Chathuranga, Thushara Balasuriya, Rasika Perera

**Affiliations:** ^1^Medical Laboratory Sciences Unit, Department of Allied Health Sciences, Faculty of Medical Sciences, University of Sri Jayewardenepura, Gangodawila, 10250 Nugegoda, Sri Lanka; ^2^Department of Biochemistry, Faculty of Medical Sciences, University of Sri Jayewardenepura, Gangodawila, 10250 Nugegoda, Sri Lanka

## Abstract

Anaemia is a major public health problem that has affected around 25% of the world's population. An analytical cross-sectional study was performed on 313 female undergraduates residing in hostels of University of Sri Jayewardenepura, Sri Lanka, during year 2011. Objective of this study was to determine prevalence and contributing factors to anaemia among the study population. Haemoglobin concentration was assayed using cyanomethaemoglobin method. A pretested self-administered questionnaire was used to retrieve information regarding dietary habits and personal factors of participants. Descriptive statistical methods, chi-square test, and independent sample* t*-test were used to analyze data. Of the 302 females, 17.5% (*n* = 53) had mild anaemia and 7.9% (*n* = 24) had moderate anaemia. Severely anaemic individuals were not observed. Participants' dietary habits and personal factors were not significantly associated with prevalence of anaemia (whether a participant is a vegetarian or not (*P* = 0.525), drinking tea within one hour of a meal (*P* = 0.775), frequency of consumption of red meat, fish, and eggs (*P* = 0.499), antihelminthic treatment within past year (*P* = 0.792), and menorrhagia (*P* = 0.560)). Anaemia in the study population is below the average for Sri Lankan data. Diet and selected medical conditions were not a causative factor for anaemia in this population.

## 1. Introduction

Anaemia is a global public health problem. It causes human death as well as social and economic problems in both developing and developed countries. According to the World Health Organization (WHO), it has affected 24.8% of the world's population [1]. In neighboring India, one in every two women suffers from anaemia [2]. When anaemia prevalence is 20–39.9% of the general population, it is considered as a moderate public health problem by WHO [1]. In Sri Lanka, anaemia has become a moderate public health problem among preschool, nonpregnant, and pregnant populations as the prevalence is 33%, 39%, and 34%, respectively [3]. According to the WHO, the highest number of individuals affected by anaemia is observed in nonpregnant women aged 15–49.99 years [4]. In Sri Lanka, 39% of females in this category are affected by anaemia [3]. Most of the anaemic cases are due to nutritional deficiencies [4].

Women of childbearing age are having an additional risk of developing anaemia because of their monthly menstrual blood loss and nearly 50 percent of females in this age group are anaemic [5]. On average a healthy woman loses about 25–30 mL of blood monthly. Therefore, the body needs to produce blood in order to compensate for this loss and if the essential nutrients required for haemopoiesis are not supplied in their diet, anaemia will develop. Prevalence of anaemia among nonpregnant women is 30.2% worldwide and in Asia it is 33% accounting to about 318.5 million individuals. Out of the total nonpregnant anaemic individuals of the world, nearly 3/4 reside in Asia. Anaemia among nonpregnant women has become a public health problem in 191 countries out of the 192 member countries of WHO [4].

The objective of this study was to determine the prevalence and contributing factors to anaemia among female undergraduates residing in the hostels of University of Sri Jayewardenepura, Sri Lanka. We assumed that the nutrient intake of female undergraduates who reside in the hostels of University of Sri Jayewardenepura is lower than that of the general population because they buy their meals from the canteens in the university premises or from the nearby food stalls. These places sell food for a considerably lower price and therefore quantity and the quality of this food items are very poor. Thus, these female undergraduates may not obtain nutrients to meet the requirement of the body and were likely to have a higher risk of developing anaemia. In turn, nutrient deficiency anemia may reduce these undergraduates' work capacities and that will adversely affect their academic performances [6].

## 2. Materials and Methods

An analytical cross-sectional study was performed to determine the proportion and contributing factors to anaemia among female undergraduates residing in hostels situated inside the premises of University of Sri Jayewardenepura, Gangodawila, Nugegoda, Sri Lanka.

### 2.1. Ethics Statement

Ethical clearance for the study was obtained from Ethical Review Committee, Faculty of Medical Sciences, University of Sri Jayewardenepura, and the study protocol was conducted according to the guidelines of the declaration of Helsinki.

A consent form along with an information sheet giving details of the study (nature of the study, what will be expected from the participants, and expected risks and benefits) were provided to all female undergraduates who were randomly selected to the sample. The details were also explained verbally to the potential participants. Afterwards, female undergraduates who provided written consent were included in the study.

### 2.2. Sample Size

A simple random sample of 332 girls was drawn from the population of female undergraduates residing in the hostels situated inside the university premises. Random numbers were generated by using blind draw method.

#### 2.2.1. Exclusion Criteria

Female undergraduates with the presence of a past history of haematological disorders (i.e., thalassaemia trait, sickle cell trait, and malignant conditions) or HIV status were excluded from the study.

### 2.3. Methods

#### 2.3.1. Variables

The study used several variables to determine the proportion and contributing factors to anaemia among female undergraduates residing in hostels situated inside the premises of University of Sri Jayewardenepura.

The variables of the study and the methods of analysis are briefly described in the following section.

### 2.4. Dependent Variables

#### 2.4.1. Severity of Anemia

The subjects who have a blood haemoglobin concentration of 11 g/dL or above were categorized as nonanaemic and subjects who have less than 11 g/dL of blood haemoglobin concentration were categorized as anaemic [3]. The anaemic individuals were further classified as mildly anaemic (haemoglobin concentration between 10.0 and 10.9 g/dL), moderately anaemic (haemoglobin concentration between 8.0 and 9.9 g/dL), and severely anaemic (haemoglobin concentration below 7.9 g/dL) [3].

A total volume of 2 mL of venous blood was obtained from each participant into EDTA (ethylenediaminetetraacetic acid) containers for haemoglobin measurement. Blood was drawn by skilled personal. Universal precautions were followed during blood collection, transportation, storage, and disposal to protect the participants as well as the researchers.

Blood haemoglobin concentrations of the participants were measured using DiaSys (diagnostic reagent for quantitative in vitro determination of haemoglobin in whole blood on photometric systems—cyanomethaemoglobin method). 20 *μ*L of blood was mixed with 5.0 mL of DiaSys solution and was kept at room temperature for 5 minutes. Then the absorbance of this mixture was measured by using a spectrophotometer at 540 nm wavelength. This absorbance value was multiplied by the calibration factor to find the haemoglobin concentration of a particular subject.

Standard haemoglobin reagent Labtest (reagent for standardization of haemoglobin) was used to calculate the calibration factor in order to convert the absorbance values into concentration values.

### 2.5. Independent Variables

A self-administered questionnaire was provided to the participants to obtain data regarding their dietary habits and personal factors as independent variables.

Dietary habits of the female undergraduates include whether subject is a vegetarian or not (no, yes), consumption of extra meals (no, yes), consumption of tea within one hour of a meal (no, yes), and number of meals supplemented with meat, fish, or egg per day (zero, one, two, and three).

Personal factors of the female undergraduates include taking antihelminthic treatment within the past year (no, yes), subject's awareness of anaemia (no, yes), and passage of clots in menstrual blood (no, yes).

Both questionnaire and blood sample of a particular individual were labeled with same reference number, so that participants could be traced at the end of the data analysis.

### 2.6. Statistical Analysis

Data were double entered and analyzed using statistical package for social sciences (SPSS) version 15. Descriptive statistical methods were used to calculate the mean values of menstrual period, age, haemoglobin concentration of anemic subjects, and haemoglobin concentration of nonanaemic subjects. Chi-square test was used to determine the difference between anaemic and nonanaemic groups in dietary habits and other personal factors of the study sample.

## 3. Results

313 female undergraduates from five hostels situated inside the university premises participated in this study. Nine incomplete questionnaires and two clotted blood samples were rejected and therefore data from 302 individuals were used in the analysis. Participants' age ranged from 20 years to 27 years and the mean age was 22.16 (±1.65) years.

### 3.1. The Proportion of Anaemic Subjects

According to this study, 17.5% (*n* = 53) had mild anaemia (haemoglobin concentration between 10.0 and 10.9 g/dL) and 7.9% (*n* = 24) had moderate anaemia (haemoglobin concentration between 8.0 and 9.9 g/dL). Severely anaemic individuals (haemoglobin concentration below 7.9 g/dL) were not observed during the study (Table 1). Blood haemoglobin levels of the study group ranged from 8.45 to 15.73 g/dL. Mean haemoglobin level of the anaemic individuals was 10.22 g/dL and the mean haemoglobin level of the nonanaemic individuals was 12.23 g/dL. There was a statistically significant difference between these two means according to the independent sample* t*-test.

### 3.2. Dietary Habits of the Study Sample

A questionnaire was used to gather information regarding the dietary habits of the female undergraduates. There were 16 (5.3%) vegetarians among the participants. Majority of the sample consume fish, meat, or egg twice a day. 83.4% of the participants take extra meals between their three main meals. There were 30 (9.9%) individuals who drink tea within one hour of a meal as a habit.

### 3.3. Personal Factors of the Study Sample

Participants' awareness about anaemia showed a statistically significant (*P* < 0.05) association with the faculty in which they are studying (Table 2). However, the proportion of anaemia was not significantly associated with the awareness of the participants.

Mean number of days that the nonanaemic subjects had their menstrual blood flow was 4.18 days and for anaemic subjects this was 4.32 days. There was no statistically significant difference between the two groups when independent sample* t*-test was performed. According to data gathered, menstrual blood of 47% of subjects (*n* = 142) contained clots. 79.5% of the subjects (*n* = 240) have taken antihelminthic treatment within past year.

Proportion of anaemia in the study group did not show a statistically significant association (*P* > 0.05) with these dietary habits and selected personal factors (Table 3).

## 4. Discussion

According to WHO data, the prevalence of anaemia in this age group in Asia and in Sri Lanka is 33% and 31.6%, respectively. But according to a survey done in Sri Lanka, the prevalence of anaemia among nonpregnant females is 39%, whereas the prevalence is 32% among 20–29-year-old nonpregnant females [3]. When we consider the above statistics, the importance of screening for anaemia among the above population is clearly evident, especially because included in the above age group are the female university undergraduates whose academic performance may be adversely affected due to anaemia. According to WHO, global anaemia prevalence among nonpregnant females is 30.2% and the proportion that we found does not significantly differ from this value (*P* = 0.075). When compared with the WHO data available on anaemia for Sri Lanka for nonpregnant females, the proportion of anaemia found in this research is at a lower value (WHO prevalence 31.6%) and our value significantly differs from WHO prevalence (*P* = 0.022) [4]. This prevalence is also lower than the prevalence among nonpregnant females of 20–29 years of age (32%) as found in an island wide research conducted by the Department of Census and Statistics in year 2007 yet the difference is not statistically significant (*P* = 0.12) [3].

The mean haemoglobin value of the study sample was 11.71 g/dL (±1.19). It was 10.22 g/dL among the anaemic participants and 12.23 g/dL among nonanaemic participants. These two figures are statistically significant according to the independent sample* t*-test. However, a research done at the University of Sharjah reported a mean Hb of 12.5 g/dL, which exceeded the mean which we acquired [7]. Also a research done in Dubai, UAE, among Medical College girls, reported a higher mean Hb value of 12.83 g/dL [8]. However, our mean Hb value is comparatively higher than that of Indian nursing students (10 g/dL ± 1.47) [9].

According to this research, over two-thirds of the anaemic students (68.83%) (*n* = 53) suffer from mild anaemia. A research conducted at the Vadodara nursing school in India, among female students within the age limit of 17–21 years, it was revealed that the prevalence of mild and moderate anaemia was almost equal (mild = 42.5%, moderate = 43.11%). Also it revealed that a higher percentage of female students were anaemic (86.6%) [9].

Food habits, which were considered in this study, including drinking tea after main meals and the frequency in which fish and meat products were consumed, did not show a statistically significant association with anaemia.

A research has been carried out in a Bangladesh university in year 2011, based on a similar hypothesis as our research, has found a higher anaemia prevalence of 55.3% and majority of them are girls (63.3%). They have also observed a significant higher proportion of male students who were unaware of anaemia [10]. In our research, there was no significant relationship between the awareness on anaemia and anaemic proportion.

A similar research carried out in the Kingdom of Saudi Arabia in King Abdulaziz University has revealed the prevalence of anaemia to be 26% among female university students [11]. Also 26.7% of anaemia prevalence was found among female students in a research carried out in the University of Sharjah [7]. In a research carried out on nonpregnant women in University of Peshawar in Pakistan, the anaemia prevalence was found to be 23.9%. [12]. Thus, the inference is that the anaemic proportion found in our research does not have a statistically significant difference from data obtained in King Abdulaziz University, University of Sharjah, and University of Peshawar (respective *P* values = 0.84, 0.63, and 0.51).

Anaemia can be caused by drinking tea after main meals. This is caused by the chemical “tanin” in tea, which reduces the absorption of iron from food [13]. However, the finding of this study as well as a study conducted in the Abdulaziz University did not show a statistically significant association between drinking tea and anaemia.

In our study the mean number of days that the nonanaemic subjects had their menstruation was 4.18 days and for nonanaemic it was 4.32 days. There was no statistically significant difference between these two values. Heavy menstrual blood loss is an important risk factor to develop iron deficiency anaemia but in our study there was no statistically significant relationship between anaemia and the number of days participants have menstruated. In this study, the number of anaemic subjects (*n* = 34) who had clots in menstrual blood was lower than the number of anaemic subjects who did not have clots in menstrual blood (*n* = 43). The research at the king Abdulaziz University too revealed that there was no significant relationship between anaemia and clots in the menstrual blood [11]. The study at University of Hail revealed that there was no relation between duration of menstruation cycle and anaemic and nonanaemic groups [14]. However, another research done among females of childbearing age in King Khalid University Hospital in Riyadh had identified menstrual blood loss lasting for more than eight days or a heavy menstrual cycle as risk factors for iron deficiency anaemia [15].

## 5. Conclusions

Anaemia in the study population is below the average for Sri Lankan data. Yet nearly one in every four female undergraduates was found to be anaemic and these results warrant serious attention to be paid regarding anemia among this population because, among other things, their academic performance may be adversely affected due to anaemia.

## Figures and Tables

**Table 1:** Proportion of participants with the four sets of classification of anaemia.

	Number	Percentage
Nonanaemic (haemoglobin 11 g/dL and above)	225	74.5%
Mild anaemic (haemoglobin level between 10 and 10.99 g/dL)	53	17.5%
Moderately anaemic (haemoglobin level between 8.0 and 9.9 g/dL)	24	7.9%
Severely anaemic (haemoglobin level below 7.99 g/dL)	0	0

Total	302	100%

**Table 2:** Subjects' awareness on anaemia according to the faculty in which they are studying.

Name of the faculty	Subject's awareness of anaemia		*P* value
	Yes	No	
Faculty of Medical Sciences	55 (100%)	0	0.000
Faculty of Humanities and Social Sciences	34 (29.3%)	82 (70.7%)	
Faculty of Management Studies and Commerce	34 (30.9%)	76 (69.1%)	
Faculty of Applied Sciences	17 (81.0%)	4 (19.0%)	

**Table 3:** Distribution of nonanaemic and anaemic subjects according to dietary habits and selected medical conditions.

	Nonanaemic (*n* = 225)		Anaemic (*n* = 77)		*P* value
	*n*	%	*n*	%	
Consumption extra meals					
Yes	193	85.8	59	76.6	0.062
No	32	14.2	18	23.4	
Vegetarian or not					
Yes	13	5.8	3	3.9	0.525
No	212	94.2	74	96.1	
Number of meals supplemented with meat, fish, or egg per day					
Zero	14	6.2	2	2.6	0.499
One	57	25.3	24	31.2	
Two	129	57.3	44	57.1	
Three	25	11.1	72	9.1	
Drinking tea within one hour of a main meal					
Yes	23	10.2	7	9.1	0.775
No	202	89.8	70	90.9	
Subject's awareness about anaemia					
Yes	101	44.9	39	50.6	0.382
No	124	55.1	38	49.4	
Passage of clots in menstrual blood					
Yes	108	48.0	34	44.2	0.560
No	117	52.0	43	55.8	
Antihelminthic treatment within the past year					
Yes	178	79.1	62	80.5	0.792
No	47	20.9	15	19.5	
